# Arterial Stiffness and Cognitive Impairment in Patients With Arterial Hypertension: CAVI and haSTART Index

**DOI:** 10.1155/ijvm/9456777

**Published:** 2026-07-28

**Authors:** T. A. Safronova, S. E. Mukhanova, T. S. Vargina, A. I. Tarzimanova, A. E. Pokrovskaya, E. R. Konchenko, A. G. Cherepanov, V. I. Podzolkov

**Affiliations:** ^1^ Sechenov First Moscow State Medical University (Sechenov University), Moscow, Russia, mma.ru

**Keywords:** arterial stiffness, CAVI, cognitive impairment, haSTART index, hypertension

## Abstract

**Introduction:**

Increased arterial stiffness is recognized as one of the established risk factors for cardiovascular diseases (CVDs), including the onset of cognitive impairment (CogI) and vascular dementia.

**Aim:**

The objective of our study was to investigate the relationship between the cardio‐ankle vascular index (CAVI) and the STELARI heart–ankle STiffness of ARTeries (haSTART) index in patients diagnosed with arterial hypertension (HTN) devoid of comorbidities. The secondary aim of the study was to explore the association of these indices with CI in patients with HTN.

**Methods:**

Arterial stiffness was assessed using CAVI and the haSTART index. Cognitive status of all patients was obtained via the Montreal Cognitive Assessment (MoCA).

**Results:**

A total cohort of 128 patients (55 men and 73 women, aged 35–79 years) was incorporated into the single‐center study, comprising 96 subjects with essential HTN (Group 1) and 32 with no HTN in history (Group 2—controls). A robust correlation was observed upon examining CAVI and the haSTART index in all subjects (*r* = 0.977, *p* < 0.001). Moreover, significant correlations were identified between the MoCA results and CAVI (*r* = −0.502, *p* = 0.001) and the haSTART index (*r* = −0.512, *p* = 0.001).

**Conclusion:**

The results of the study indicated a relationship between arterial stiffness and CI, as evidenced by the association between CAVI and MoCA results. Furthermore, we identified for the first time a correlation between the haSTART index and MoCA, thereby reflecting the association between arterial stiffness and CI.

## 1. Introduction

The prevalence of arterial hypertension (HTN) continues to rise, reaching 1.4 billion patients globally, as reported by the WHO for 2024 [1]. In Europe, the prevalence of HTN is observed to be between 30% and 45% within the adult population [2]. A comprehensive national population‐based study, known as “Epidemiology of Cardiovascular Diseases in Regions of the Russian Federation, Third Survey” (ESSE‐RF‐3), indicated the prevalence of HTN in Russia to be 53.9% during the period of 2020–2022 [3].

According to Russian and European clinical guidelines on HTN (the Russian Society of Cardiology [RSC] 2024 and the European Society of Hypertension [ESH] 2023), arterial stiffness is recognized as one of the proven risk factors for cardiovascular disease (CVD) [4, 5]. Long‐term HTN considerably accelerates and enhances the age‐related increase in arterial stiffness through a number of interconnected pathophysiological mechanisms: endothelial dysfunction, concentric remodeling of the arterial wall, oxidative stress, arterial calcification, and an imbalance between vasoconstrictors and vasodilators, among others [6]. HTN can hasten the degradation of elastin fibers and lead to an excess of collagen, resulting in diminished elasticity of the arterial wall and an increase in stiffness. This phenomenon may provoke a rise in blood pressure, instigating a vicious cycle that exacerbates both HTN and arterial stiffness [7]. The pathological processes associated with HTN amplify age‐related alterations in blood vessels, thereby accelerating vascular aging [8]. Vascular aging represents a generalized physiological process that influences all layers of the arterial wall, characterized by both structural (hypertrophy and fibrosis) and functional (increased pulse wave velocity [PWV], total peripheral resistance, and arterial stiffness) changes within the arteries [9].

**Figure 1:**
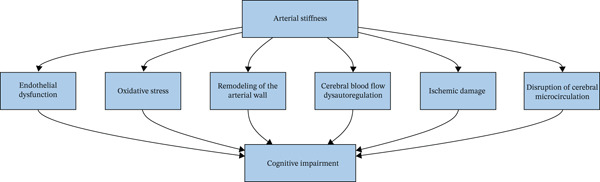
The impact of arterial stiffness on the development of cognitive functions.

According to the findings presented in “Clinical Applications Measuring Arterial Stiffness: An Expert Consensus for the Application of Cardio‐Ankle Vascular Index” (2022) [10] and “Diagnosis of Arterial Stiffness Using the Cardio‐Ankle Vascular Index. Expert Consensus” (2025) [11], increased arterial stiffness constitutes a significant risk factor for the onset of cognitive impairment (CogI), including vascular dementia. Arterial stiffness is linked to the progression of CI through multiple pathological mechanisms [12, 13]: Impaired cerebral microcirculation results in structural and functional alterations in the brain′s small vessels, thereby impairing tissue perfusion issues and disrupting metabolic processes while also increasing the permeability of the blood–brain barrier, which facilitates the migration of plasma proteins and the activation of macrophages [12]; endothelial dysfunction occurs due to disrupted synthesis of nitric oxide (NO) and other vasodilators, leading to an imbalance between vasodilatory and vasoconstrictive factors, which further deteriorates microcirculation and contributes to the development of ischemia [14]; arterial remodeling accelerates PWV, which may result in damage to cerebral vessels and diminishes the vessels′ capacity to adapt to changes in perfusion, thereby impairing cerebral blood flow [15]; and other factors (see Figure 1). Qiu and Fratiglioni concluded that dementia in the elderly is a chronic condition that manifests in midlife among patients with HTN and vascular pathology [16].

Since 2013, the measurement of PWV has been regarded as the “gold standard” for evaluating the stiffness of large vessels, as delineated in the European Society of Hypertension and the European Society of Cardiology (ESH/ESC) guidelines for the management of HTN. This criterion is included in the compilation of fundamental screening tests aimed at detecting target organ damage in HTN and is also referenced by RSC in the 2024 HTN guidelines “Arterial Hypertension in Adults” as the primary methodology for assessing arterial stiffness. The rationale behind this method is that a decrease in arterial elasticity correlates with an increase in PWV. Consequently, the investigation of the cardio‐ankle vascular index (CAVI) has garnered significance. CAVI is recognized as a validated parameter for quantifying arterial stiffness [10]. This index is computed employing volumetric sphygmography and is dependent upon PWV values. A notable advantage of this method is that the results of the measurements are unaffected by the blood pressure level at the time of assessment. The index is derived automatically, with higher values indicating increased arterial stiffness. Researchers have substantiated the prognostic significance of CAVI as a cardiovascular risk marker in European populations [10] and among residents of Japan [17]. Furthermore, within the Russian population, the survey ESSE‐RF assessed vascular wall conditions utilizing volumetric sphygmography on the VaSera VS‐1500 device (Fukuda Denshi, Japan), thereby affirming the substantial potential of CAVI in detecting asymptomatic damage to the walls of major vessels [18]. The authors have asserted that this methodology may be employed to ascertain cardiovascular risk among patients with HTN.

Recently, the STELARI heart–ankle STiffness of ARTeries index (START index, haSTART index) has been subjected to considerable research—a novel Russian metric for assessing arterial stiffness [19]. The haSTART index is characterized by its omission of empirical coefficients in its calculations, which presents a notable advantage over the CAVI metric.

START=−lnPs/Pdvs−U+lnPs/Pdvs−U2−vsvs−Uln2Ps/Pd1−a1−avs,

where *P*_*d*_ is the diastolic blood pressure (DBP); *P*_*s*_ is the systolic blood pressure (SBP); *v*_*s*_ is the maximum systolic blood flow velocity; *U* is the rupture velocity, which aligns with the previously measured PWV; *a* is the ratio of *v*_*s*_ to *v*_*d*_; and *v*_*d*_ is the end‐diastolic blood flow velocity.

The variation in the velocity of pulse wave propagation through various types of arteries, alongside the nonlinear effects that impact wave speed at elevated amplitudes, is taken into consideration. This methodology facilitates a more accurate representation of arterial stiffness [19]. A study conducted by Vasyutin et al. [20] and Sumin et al. [21] indicates that in healthy subjects, the haSTART index exhibits a significant correlation with CAVI, thereby suggesting their comparability in evaluating arterial stiffness. Several investigations have similarly demonstrated this correlation between the indices in patients with HTN, coronary heart disease, stroke, diabetes mellitus, and chronic kidney disease [22–24].

In recent years, the association between arterial stiffness and CI has been the subject of extensive research. Numerous studies [25–28] have established that increased arterial stiffness, as measured by CAVI, correlates with a decline in cognitive function, as assessed by the Montreal Cognitive Assessment (MoCA) scale. Consequently, Iulita et al. posit that increased stiffness of the arterial wall adversely affects microcirculation, particularly in the brain, contributing to the onset of CI [29]. However, a literature review reveals a lack of studies examining the relationship between cognitive functions and the haSTART index.

The objective of this study was to investigate the relationship between CAVI and the haSTART index in hypertensive patients devoid of CVD. The subsequent component of the study aimed to analyze the correlation between these indices and CI in hypertensive patients.

## 2. Methods

The study was performed in accordance with Good Clinical Practice standards and in compliance with the ethical principles delineated in the Declaration of Helsinki. The research protocol was approved by the Local Ethics Committee of the Federal State Autonomous Educational Institution of Higher Education “I.M. Sechenov First Moscow State Medical University” of the Ministry of Health of Russia (Sechenov University), as per protocol extract No. 1‐25 dated January 23, 2025.

Inclusion criteria are as follows: subjects aged 18 years or older with signed informed patient consent for participants included in the study group—essential HTN in history. Exclusion criteria encompassed secondary arterial HTN (secondary HTN), mental disorders, severe somatic conditions, oncological diseases, contraindications to volumetric sphygmography utilizing the Fukuda Denshi VS‐1500 VaSera, and absence of signed informed patient consent. Additionally, the exclusion criteria comprised an ankle–brachial index (ABI) of ≤ 0.9 and the presence of brachiocephalic artery stenosis exceeding 50%.

A total cohort of 128 patients (55 men and 73 women, aged 35–79 years) was included in the single‐center study. Ninety‐six patients were diagnosed with essential HTN (Group 1), while 32 subjects without HTN served as controls (Group 2). All patients underwent examinations in accordance with the “Arterial Hypertension in Adults” clinical guidelines (RSC 2024). Patients with HTN received either dual or triple combination antihypertensive therapy, consistent with the clinical guidelines. At the time of study entry, all patients had attained the target blood pressure values.

Blood pressure and CAVI measurements were obtained during standard clinical practice utilizing a VaSera VS‐1500 sphygmograph [17]. Subsequently, the novel haSTART index was computed based on the data acquired through volumetric sphygmography [19]. The haSTART index was calculated using an online calculator: https://stelari-start.com.

To evaluate cognitive status, all patients underwent MoCA [30]. Scores of 26 points or higher on the MoCA scale were categorized as normal, whereas a score of 25 points or lower was interpreted as indicative of CI. We have received an official certificate from MoCA Cognition authorizing the use of the MoCA in our study.

Statistical data was processed using the SPSS 22 software. The compliance of quantitative indicators with the normal distribution was assessed through the Shapiro–Wilk test. Quantitative data were characterized using the median (Me), along with the first and third quartiles [Q1; Q3]. Categorical variables are presented in terms of absolute and relative (percentage) frequencies. The Mann–Whitney test was applied for comparisons between Group 1 and Group 2. Nominal data were analyzed using Pearson′s *x* ^2^ and Fisher′s exact test. Correlation analysis was conducted employing the Spearman correlation coefficient, with a 95% confidence interval (CI). Multiple linear regression models for CAVI and the haSTART index were created. To develop these models, indicators exhibiting statistically significant correlations with these indices and the MoCA were utilized. The critical significance level was established at *p* ≤ 0.05.

## 3. Results

The clinical characteristics of the participants involved in this study are delineated in Table 1. The Me age [Q1; Q3] of subjects within the hypertensive cohort (Group 1) was 59 [52; 67] years, in contrast to 53 [48; 60] years for the Controls (Group 2); no statistically significant differences were observed (*p* = 0.063). Both groups exhibited a predominance of female participants, comprising 56.2% and 59.4%, respectively. The body mass index (BMI) in the hypertensive group was significantly higher (*p* = 0.008), recorded at 29.54 [25.97; 32.2], as compared to 25.17 [22.64; 30.28] in the control group. Furthermore, no significant differences were found between smokers and nonsmokers (*p* = 0.235).

**Table 1:** Clinical characteristics of the study groups.

*N*(%) or Ме [Q25; Q75]	HTN (96)	Without HTN (32)	*p* value
Sex, m/f	42/54 (43.8/56.2)	13/19 (40.6/59.4)	0.757
Age (years)	59 [52; 67]	53 [49; 60]	0.063
Smokers	35 (36.5)	8 (25)	0.235
BMI (kg/m^2^)	29.54 [25.97; 32.2]	25.17 [22.64; 30.28]	0.008 ^∗∗^
Hypertension grade	1—8 (8)	—	—
	2—22 (23)		
	3—66 (69)		
Hypertension stage	I—60 (62.5)	—	—
	II—36 (37.5)		
	III—0		
HTN history (years)	10 [5; 17.5]	—	—
TC (mmol/L)	5.28 [4.26; 6.01]	4.68 [4.57; 5.91]	0.669
HDL (mmol/L)	1.22 [1.03; 1.5]	1.4 [1.33; 1.46]	0.409
LDL (mmol/L)	3.33 [2.78; 4.12]	3.22 [3.03; 3.37]	0.605
VLDL (mmol/L)	0.71 [0.52; 1.03]	0.7 [0.48; 0.75]	0.665
Triglycerides (mmol/L)	1.57 [1.11; 2.38]	1.5 [1.06; 1.64]	0.624
Creatinine (*μ*mol/L)	83 [76; 97.4]	79.5 [69; 91.5]	0.187
GFR (mL/min/1.73 m^2^)	73.7 [63; 84.5]	85.5 [69.8; 93.7]	0.02 ^∗^
SBP (mmHg)	136 [123.5; 145]	126 [121.5; 133]	0.004 ^∗∗^
DBP (mmHg)	87 [79; 93]	81 [76; 86.5]	0.024 ^∗^
PP (mmHg)	50 [43; 56.5]	45 [39; 56.5]	0.274
HR (bpm)	69.5 [63; 77]	69 [61.5; 77]	0.61
ABI	1.11 [1.06; 1.17]	1.11 [1.05; 1.15]	0.517
LVMI (g/m^2^)	73.69 [63.3; 93.5]	69.52 [58.57; 89.08]	0.348
IVS (cm)	0.9 [0.8; 1]	0.8 [0.8; 0.95]	0.034 ^∗^
LVPW (cm)	0.9 [0.8; 1.0]	0.8 [0.8; 0.8]	0.086
LVEF	63 [62; 65]	65 [64; 65]	0.015 ^∗^
LVEDD (cm)	4.3 [4.2; 4.6]	4.3 [4.15; 4.7]	0.802
LVESD (cm)	2.9 [2.8; 3.1]	2.8 [2.75; 3.05]	0.398
САVI	8.35 [7.25; 9.55]	7.6 [7.1; 8.2]	0.003 ^∗∗^
haSTART	8.4 [6.8; 10.6]	7.3 [6.6; 8.35]	0.012 ^∗^
MoCA, score	25 [24; 27]	27 [26.5; 28]	0.001 ^∗∗^

Abbreviations: ABI, ankle–brachial index; BMI, body mass index; DBP, diastolic blood pressure; f, female; GFR, glomerular filtration rate; HDL, high‐density lipoprotein; HR, heart rate; IVS, interventricular septum; LDL, low‐density lipoprotein; LVEDD, left ventricular end‐diastolic diameter; LVEF, left ventricular ejection fraction; LVESD, left ventricular end‐systolic diameter; LVMI, left ventricular mass index; LVPW, left ventricular posterior wall; m, male; PP, pulse pressure; SBP, systolic blood pressure; TC, total cholesterol; VLDL, very‐low‐density lipoprotein.

^∗^ *p* value < 0.05;  ^∗∗^ *p* value < 0.01.

**Figure 2:**
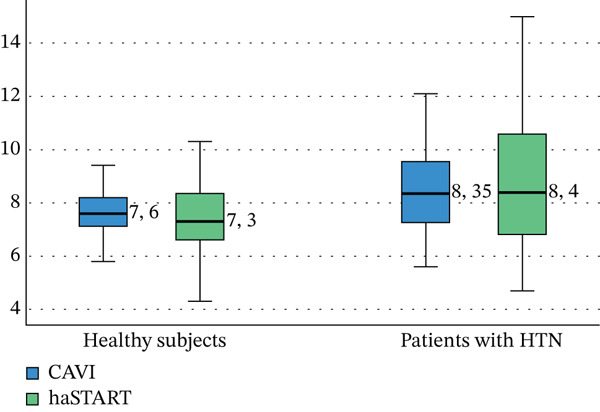
Comparison of CAVI and haSTART levels in the HTN group and the control group.

Arterial stiffness, as determined by CAVI (see Figure 2), was significantly elevated (*p* = 0.003) in Group 1, with a Me of 8.35 [7.25; 9.55], in comparison to Group 2, which exhibited a Me of 7.6 [7.1; 8.2]. Similarly, the haSTART index (refer to Figure 2) revealed comparable trends: In patients with HTN, the index indicated a Me of 8.4 [6.8; 10.6], exceeding the Me in the nonhypertensive group, which was 7.3 [6.6; 8.35]. The observed difference reached statistical significance (*p* = 0.012).

**Figure 3:**
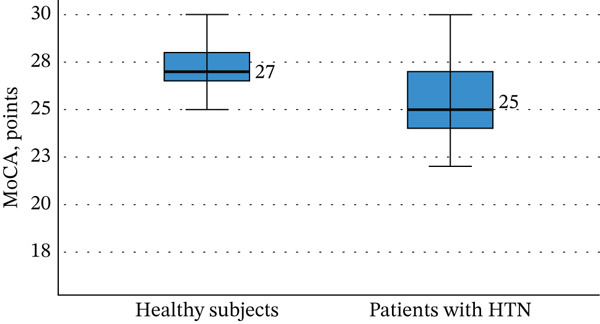
Comparative analysis of MoCA scores between the HTN group and the control group.

CI exhibited a higher prevalence among patients with HTN (*p* = 0.001), characterized by a lower MoCA score of 25 [24; 27], compared to subjects without HTN, who had an MoCA score of 27 [26.5; 28] (Figure 3). In Group 1, 52 (57.1%) patients were identified with CI, whereas in Group 2, this was observed in three (9.4%) subjects. Within the hypertensive patient cohort, mild CI was present in 50 (96.2%) patients, along with two (3.3%) exhibiting moderate CI; conversely, in the control group, three (100%) subjects had mild CI.

**Figure 4:**
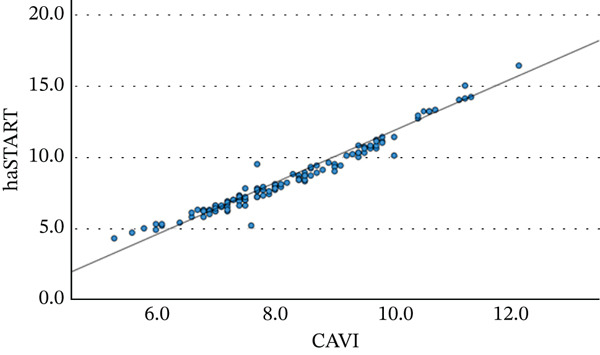
The correlation between CAVI and the haSTART index.

Furthermore, patients in Group 1, in comparison to the control group, exhibited elevated values of SBP (*p* = 0.004), DBP (*p* = 0.024), interventricular septum (IVS) thickness (*p* = 0.034), and diminished values of glomerular filtration rate (GFR) (*p* = 0.02) and left ventricular ejection fraction (LVEF) (*p* = 0.015). When examining the arterial stiffness indicators, CAVI and haSTART (Figure 4), a robust correlation was observed among all subjects (*r* = 0.977, *p* < 0.001), with a more pronounced dependence evident in female subjects (*r* = 0.975, 95% CI 1.643; 1.833, *p* < 0.001), as compared to their male counterparts (*r* = 0.686, 95% CI 1.367; 2.496, *p* < 0.001). A stronger association between these indices was identified in patients diagnosed with HTN (*r* = 0.982, 95% CI 1.712; 1.851, *p* < 0.001) relative to the control group (*r* = 0.613, 95% CI 1.414; 4.028, *p* < 0.001).

Utilizing Spearman′s correlation analysis in the examination of various indicators in relation to CAVI and the haSTART index, significant correlations of moderate strength were identified with age (CAVI *r* = 0.639, *p* = 0.001; haSTART *r* = 0.616, *p* = 0.001), LVMI (CAVI *r* = 0.359, *p* = 0.014; haSTART *r* = 0.432, *p* = 0.003), LVPW (CAVI *r* = 0.325, *p* = 0.027; haSTART *r* = 0.351, *p* = 0.017), and LVEDD (CAVI *r* = 0.357, *p* = 0.015; haSTART *r* = 0.422, *p* = 0.003).

A significant correlation was also observed for CAVI with PP (*r* = 0.289, *p* = 0.05) and for the haSTART index with the GFR indicator (*r* = −0.312, *p* = 0.035). Furthermore, for the MoCA results, significant correlations were identified with both CAVI (*r* = −0.502, *p* = 0.001) and the haSTART (*r* = −0.512, *p* = 0.001) index, in addition to age (*r* = −0.465, *p* = 0.001), LVEDD (*r* = −0.317, *p* = 0.032), and HDL (*r* = 0.365, *p* = 0.013).

Following an analysis of the correlation relationships, we identified the indicators that were statistically significant and employed them to create two multiple linear regression models for CAVI and the haSTART index. Age was excluded from the model creation for CAVI and the haSTART index, as its association with CAVI and the new index had been previously demonstrated in healthy volunteers [20, 31].

### 3.1. M1: Multiple Linear Regression Model for CAVI

In the development of the multiple linear regression model for CAVI, the following variables were incorporated: LVEDD, LVMI, PP, LVPW, and MoCA. The observed relationship is articulated by the following equation:

YCAVI=7.205+0.028∗XPP+0.964∗XLVEDD+−0.184∗XMoCA,

where *Y*_CAVI_ is the CAVI; *X*_PP_ is the pulse pressure, millimeters of mercury; *X*_LVEDD_ is the left ventricular end‐diastolic diameter, centimeters; and *X*_MoCA_ is the MoCA, points.

A decline of 1 point on the MoCA scale is projected to result in an increase in CAVI of 0.184, contingent upon the constancy of the other variables. The resultant regression model is characterized by a correlation coefficient of *r* = 0.565, which indicates a moderate correlation according to the Chaddock scale. The significance level was determined to be *p* < 0.001.

### 3.2. M2: Multiple Linear Regression Model for the haSTART Index

In the development of the multiple linear regression model for the haSTART index, the following variables were incorporated: LVEDD, LVMI, GFR, LVPW, and MoCA. The delineated relationship is articulated by the following equation:

YhaSTART=11.1+3.32∗XLVPW+−0.02∗XGFR+−0.174∗XMoCA,

where *Y*_haSTART_ is the haSTART index; *X*_LVPW_ is the left ventricular posterior wall, centimeters; *X*_GFR_ is the GFR, mL/min/1.73 m^2^; and *X*_MoCA_ is the MoCA, points.

A reduction of 1 point in the MoCA score is supposed to result in an increase of 0.174 in the haSTART index, assuming other variables remain constant. The derived regression model exhibits a correlation coefficient of *r* = 0.633, which is indicative of a moderate strength of correlation as delineated by the Chaddock scale. The significance level was determined to be *p* < 0.001.

## 4. Discussion

The primary objective of the present study was to assess the relationship between CAVI and the haSTART index in patients with arterial HTN. A strong correlation between these indices was observed in both men and women, regardless of HTN status (*p* < 0.001). Correlation analysis indicated that both indices exhibited similar relationships with age, LVEDD, LVMI, LVPW, and MoCA. Additionally, PP was found to be significant for CAVI, whereas GFR was significant for the haSTART index. Our research demonstrated a positive correlation between CAVI and LVMI, a finding that has been corroborated by other studies [5, 10, 23]. A similar correlation was established for the haSTART index.

In this study, lipid profile parameters did not show an association with CAVI and the haSTART index, contrasting with findings from other studies [32, 33]. This discrepancy may be attributed to the inclusion of patients without significant atherosclerosis in our cohort. Among healthy subjects, a study conducted by Sumin et al. [21] identified statistically significant correlations among the haSTART index, age, and GFR; notably, our results echoed these findings in hypertensive patients. Furthermore, another study by Sumin et al. [23], which examined CAVI and the haSTART index in hypertensive patients with CVD, retained a statistically significant association between SBP and CAVI (*p* = 0.02) within regression models. Conversely, such an association was not established for the START index (*p* = 0.09), aligning with our data. A large prospective study [34] indicated that SBP significantly influences arterial stiffness as measured by CAVI, whereas SBP exerted a comparatively lesser influence. In a study conducted by Vasyutin et al. [20] with 928 participants, multiple linear regression analysis revealed statistically significant correlations between CAVI and the haSTART index with DBP and PP. However, our findings indicated that PP was the variable that significantly correlated with CAVI.

The resulting multiple linear regression models for CAVI (*Y*_CAVI_) and the haSTART (*Y*_haSTART_) index suggest that arterial stiffness, as measured by these indices, is significantly more influenced by structural changes (LVEDD, LVMI, and LVPW) than by hemodynamic parameters (SBP, DBP, and PP). The regression models also indicated a significant relationship between cognitive function and arterial stiffness measured using both CAVI and the haSTART index. Previous studies have established that elevated CAVI levels are a risk factor for the development of CVD and cognitive impairment, including dementia [27, 28]. Our results are consistent with those findings, highlighting the relationship between arterial stiffness and mild CI in both hypertensive patients and healthy controls. A limited number of studies [35, 36] have failed to find an association between arterial stiffness and CI; however, these investigations used PWV and ABI as markers of arterial stiffness, as well as neurocognitive scales other than MoCA. To date, we are unaware of any studies that explore the relationship between CI and the haSTART index.

## 5. Conclusion

The pronounced correlation between the haSTART index and CAVI (*r* = 0.977, *p* = 0.001) indicates that the haSTART index may serve as a complementary tool to CAVI in analogous clinical contexts. In subjects diagnosed with HTN, the haSTART index has demonstrated a correlation with target organ damage, akin to that observed with CAVI. Further investigation into the haSTART index across various pathologies, in addition to prospective observations, is necessary to evaluate the prognostic significance of this novel index.

The findings of the study revealed an association between arterial stiffness and CI, particularly as evidenced by the relationship between CAVI and results from MoCA. Notably, our data illustrate a connection between arterial stiffness and mild CI in patients presenting with HTN who exhibit no substantial atherosclerosis of the brachiocephalic arteries, as well as in healthy subjects. This research represents the inaugural demonstration of a relationship between the novel haSTART index and CI as quantified through the MoCA.

## Author Contributions

V.I.P.: conceptualization, methodology, supervision, validation, and writing—review and editing. T.A.S.: data analysis, writing—original draft, and writing—review and editing. A.I.T.: formal analysis and writing—review and editing. T.S.V.: literature search and writing—review and editing. A.E.P.: methodology and writing—review and editing. S.E.M.: patient recruitment, data collection, and writing—original draft. A.G.C.: statistical advice, translating, and culturally adapting the source content. E.R.K.: database development.

## Funding

No funding was received for this manuscript.

## Ethics Statement

The research protocol was approved by the Local Ethics Committee of the Federal State Autonomous Educational Institution of Higher Education “I.M. Sechenov First Moscow State Medical University” of the Ministry of Health of Russia (Sechenov University), as per protocol extract No. 1‐25 dated January 23, 2025. The procedures used in this study adhere to the tenets of the Declaration of Helsinki.

## Consent

Informed consent was obtained from all individual participants included in the study.

## Conflicts of Interest

The authors declare no conflicts of interest.

## Data Availability

The data that support the findings of this study are available from the corresponding author upon reasonable request.
